# Mechanisms underlying the therapeutic effects of Gang Huo Qing wen granules in the treatment of influenza based on network pharmacology, molecular docking and molecular dynamics

**DOI:** 10.1038/s41598-024-62469-2

**Published:** 2024-07-09

**Authors:** Wenyu Wu, Wanning Lan, Xin Jiao, Axue Shao, Peng Wu, Kai Wang, Shaofeng Zhan

**Affiliations:** 1 The First Clinical Medical School of Guangzhou University of Chinese Medicine, Guangzhou, China; 2 The First Affiliated Hospital of Guangzhou University of Chinese Medicine, Guangzhou, China

**Keywords:** *Ganghuo Qingwen Granules (20g/time, 3 times/day)*, Influenza, Network pharmacology, Molecular docking, Molecular dynamics simulation, Mechanism, Computational biology and bioinformatics, Drug discovery

## Abstract

Influenza (Flu) is a severe health, medical, and economic problem, but no medication that has excellent outcomes and lowers the occurrence of these problems is now available. *GanghuoQingwenGranules* (*GHQWG*) is a common Chinese herbal formula for the treatment of influenza (flu). However, its methods of action remain unknown. We used network pharmacology, molecular docking, and molecular dynamics simulation techniques to investigate the pharmacological mechanism of *GHQWG* in flu. TCMSP and various types of literature were used to obtain active molecules and targets of *GHQWG*. Flu-related targets were found in the Online Mendelian Inheritance in Man (OMIM) database, the DisFeNET database, the Therapeutic Target Database (TTD), and the DrugBank database. To screen the key targets, a protein–protein interaction (PPI) network was constructed. DAVID was used to analyze GO and KEGG pathway enrichment. Target tissue and organ distribution was assessed. Molecular docking was used to evaluate interactions between possible targets and active molecules. For the ideal core protein–compound complexes obtained using molecular docking, a molecular dynamics simulation was performed. In total, 90 active molecules and 312 *GHQWG* targets were discovered. The PPI network's topology highlighted six key targets. *GHQWG*'s effects are mediated via genes involved in inflammation, apoptosis, and oxidative stress, as well as the TNF and IL-17 signaling pathways, according to GO and KEGG pathway enrichment analysis. Molecular docking and molecular dynamics simulations demonstrated that the active compounds and tested targets had strong binding capabilities. This analysis accurately predicts the effective components, possible targets, and pathways involved in *GHQWG* flu treatment. We proposed a novel study strategy for future studies on the molecular processes of *GHQWG* in flu treatment. Furthermore, the possible active components provide a dependable source for flu drug screening.

## Introduction

Influenza is a seasonal acute respiratory infection of variable severity caused by infection with negative-stranded RNA influenza viruses of the Orthomyxoviridae family. According to the antigenicity of the viral nucleoprotein NP and matrix protein M1, influenza viruses can be categorized into influenza A (IAV), B (IBV), C (ICV), and D (IDV)^1^, and the main types of influenza viruses that infect humans are A, B, and C. IAV has a wide range of hosts which can be hosted on humans, birds, and other mammals. It is highly sensitive to antigenic variation and able to mutate through a variety of pathways, among which the antigenic drift mechanism can disrupt the protective effect of antibodies on organisms, resulting in annual influenza epidemics; whereas antigenic shift can change the biology of viruses, producing viruses with different antigenic properties, and when new strains with significant antigenic differences emerge, they may be able to form a worldwide pandemic^1,2^.

The clinical symptoms of influenza are dominated by acute high fever, generalized pain, malaise and other systemic toxic symptoms and respiratory symptoms such as cough and sore throat, and most patients with influenza can recover within 1 week. Although it is a self-limiting disease, for the elderly and frail patients and patients with other underlying diseases, it can cause a variety of complications, develop into severe influenza, induce multiple organ failure, and ultimately lead to death. The high variability, high drug resistance and strong infectivity of influenza viruses pose a great challenge to global public health^3^. In 2019, WHO reported that there were nearly 1 billion cases of influenza worldwide each year, of which 3 to 5 million were severe cases and 290,000–650,000 were fatal cases^4^. It can be seen that influenza is characterized by high contagiousness, high morbidity, and high morbidity and mortality.

At present, the treatment methods of Western medicine include vaccine prevention, prompting interruption of virus transmission and symptomatic treatment after infection. However, there are shortages of vaccines, inadequate coverage, and their effectiveness is often affected by a number of factors, such as age, immune function, and the viral strain^2,5^. On the one hand, age and immunocompromised people are at higher risk of complications from vaccination. On the other hand, influenza viruses are highly susceptible to mutation, and it is clearly unlikely that a new vaccine can be developed in a short period of time in the face of a new influenza virus. This leads to a lag in vaccine development relative to influenza outbreaks. Current antiviral drugs are mainly neuraminidase inhibitors (oseltamivir and zanamivir), cap-dependent endonuclease inhibitors (baloxavir maboxir), and adamantanes (e.g., amantadine and amantadine), the former of which are active against both IAV and IBV, and the latter of which is only active against IAV and are not recommended for influenza^6^. In addition, antiviral drugs also face a number of other challenges, such as increased drug resistance, controversial prognostic improvement, and efficacy affected by the time of drug initiation. In summary, influenza viruses are fast-mutating and have a high potential for transmission from animal hosts to humans, so they can emerge or re-emerge as “new viruses” with the potential to spread rapidly among susceptible populations and cause global epidemics or pandemics. However, the existing means of preventing and treating influenza are extremely limited, so it is crucial to find a new drug or vaccine that can inhibit the replication and spread of the virus in a timely manner, alleviate the symptoms and minimize the occurrence of complications.

Traditional Chinese medicine (TCM) has been widely used in China and some other nations for more than 2000 years to treat a variety of infectious disorders, including COVID-19 and prior malaria^7^. TCM has contributed to the prevention and control of significant infectious disease epidemics and has emerged as a key component and focal point in China^8^. The pathophysiology of influenza disease is characterized by “dampness, heat, poison, and stasis” and is referred to in TCM theory as “YI Disease,” which is produced by “YI QI^9^.” Zhang Zhongjing created the Shang Han hypothesis, also known as the “Treatise of Exogenous Febrile Diseases” or “Discourse on Cold-Damage Disorders,” during the Han dynasty to help cure influenza. The development of “Wen Yi Lun (Analysis of Epidemic Warm Diseases)” in the Ming and Qing Dynasties later revealed that TCM had made significant contributions to the prevention and treatment of influenza for thousands of years^10,11^. Previously, TCM demonstrated a positive anti-viral effect on the influenza virus and alleviated the symptoms among patients^12,13^. *Ganghuo Qingwen Granules (20g/time, 3 times/day)* (*GHQWG*) is an empirical prescription in the First Affiliated Hospital of Guangzhou University of Chinese Medicine and has shown significant clinical efficacy against influenza (flu), mainly comprised of *Ilex asprella root* (*Gangmeigen, GMG*), *Pogostemon cablin* (*Guanghuoxiang, GHX*), *Lonicerae Japonicae Flos* (*Jinyinhua, JYH*), *Forsythiae Fructus* (*Lianqiao, LQ*), *Saposhnikoviae Radix* (*Fangfeng, FF*), *Notopterygii Rhizoma Et Radix* (*Qianghuo, QH*), *Radix Bupleuri* (*Chaihu, CH*)*, Atractylodes lancea* (*Cangzhu, CZ*), *Schizonepetae Spica* (*Jingjiesui, JJS*) and *Bovis Calculus* (*Niuhuang, NH*), which has the efficacy of “dispersing the wind and relieving epidemiology, clearing heat and detoxifying toxins, and dispelling dampness and neutralizing the effect of wind-heat and dampness,” and it is commonly used in the clinical diagnosis and treatment of the epidemic flu with the wind-heat and dampness syndrome. Previous studies have indicated that *GHQWG* could remarkably reduce the exudation of inflammatory mediators in mice with H1N1 (A/FM1/1/47) influenza virus pneumonia by improving anti-inflammatory cytokine levels (IFN-γ, TNF-α, IL-2), lowering pro-inflammatory cytokines (IL-5, IL4)^14^. However, systematic studies of the mechanisms by which *GHQWG* achieves its anti-flu properties, including evaluations of putative targets, cellular processes, and metabolic pathways, are missing.

Complex chemicals used in TCM have numerous clinical targets and pathways. For mechanistic research of TCM, conventional pharmacology methodologies are so restricted^15^. A novel idea known as "network pharmacology," put forth by Shao Li in 2013, offered a fresh method for figuring out how TCM formulas work^16^. The development of bioinformatic networks and network topology investigations are part of TCM network pharmacology, which also encompasses virtual computing, high-throughput data processing, and network database retrieval^17^. This method is appropriate for studying TCM because it focuses on multi-component, multi-channel, and multi-target synergy^18,19^. Network pharmacology, which is based on computational prediction, has recently merged with bioinformatics, making it a reliable way to systematically disclose the biological mechanisms behind complex diseases and therapeutic actions at the molecular level^20,21^. More research is being done using network pharmacology to determine the mechanisms underlying TCM's therapeutic benefits on diverse ailments^22,23^.

In 2007, Hopkins, a British pharmacologist, first proposed the concept of network pharmacology (NP), which is defined as a branch of pharmacology based on the theoretical foundation of systems biology and multidirectional pharmacology, and utilizing the method of biomolecular network analysis to select specific nodes for new drug design and target analysis^18,24^. Molecular docking is a computer-aided drug design (CADD) method^25^, which is an important tool in NP to predict the binding affinity between drugs and targets. They can predict the binding affinity between a drug and its target by constructing drug–target-pathway network models and simulating drug–target interactions, which can reveal the mechanism of action and potential side effects of the drug. TCM is characterized by "multi-components, multi-targets, and multi-pathways", which coincides with the "multi-genes, multi-targets" pharmacological research characteristics of NP^4,26^. With the soundness of database, the maturity of CADD technology and the modernization of TCM, the research of NP and molecular docking technology in the field of TCM has become more and more extensive, including but not limited to the prediction of Chinese medicine targets, understanding of the biological basis of diseases and syndromes, the network regulation mechanism of TCM, and the identification of biomarkers of diseases and syndromes based on the biological network^5,27^, which has brought vigorous opportunities and has also brought new opportunities for the development of Chinese medicine pharmacology. It also provides new horizons for the search of new drugs against influenza virus.

**Figure 1:**
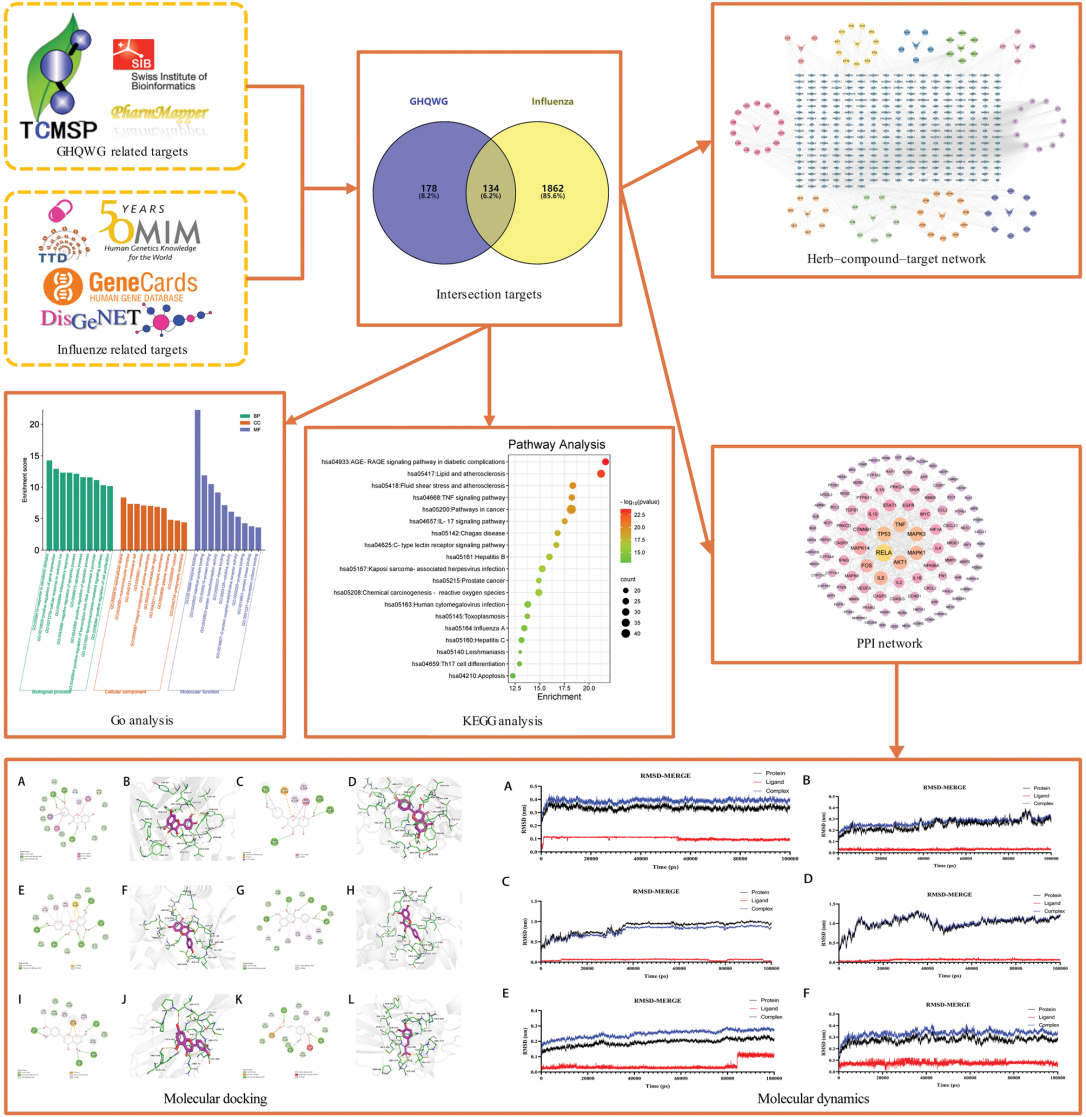
Workflow of the network pharmacological investigation strategy of *GHQWG* in the treatment of flu.

In summary, the pandemic nature of influenza, the drug resistance of the virus and the limited means currently available to Western medicine to cope with influenza have prompted researchers around the world to search for new ways to prevent and treat influenza. TCM possesses unique advantages and broad prospects in the influenza. Clinical experience has found that GHQWG can effectively inhibit the progression of the disease and improve the prognosis, but its mechanism of action still needs to be further explored. NP and molecular docking technology can be a good way to explore the mechanism of action of Chinese medicine pharmacodynamic substances and therapeutic diseases. Therefore, this research intends to search for active molecules and targets of GHQWG, influenza-related targets, construct the network system of GHQWG-influenza by NP and molecular docking techniques to predict the main bioactive substances, possible targets and signaling pathways of GHQWG in the treatment of influenza. We hope that this work will provide a scientific elucidation of GHQWG for influenza, align with the frontiers of modern science and technology, lay the foundation for further research on the mechanism of action of GHQWG for influenza, and provide new insights into the treatment of influenza by TCM for researchers around the world. The flow chart of this study is shown in Fig. 1.

## Materials and methods

### Screening and target prediction of active components of *GHQWG*

The active components of Ilex asprella root, Pogostemon cablin, Lonicerae Japonicae Flos, Forsythiae Fructus, Saposhnikoviae Radix, Notopterygii Rhizoma Et Radix, Radix Bupleuri, Atractylodes lancea, Schizonepetae Spica and Bovis Calculus in *GHQWG* were searched by application analysis platform and database system pharmacology of Chinese medicine (TCMSP, https://tcmspw.com/tcmsp.php)^28^ and the targets of active components were predicted. We screened the active ingredients that meet both oral bioavailability (OB) ≥ 30% and drug-like (DL) ≥ 0.18 by pharmacodynamics^29,30^. Since "Ilex asprella root" was not searched in the TCMSP database, the chemical composition of Ilex asprella root was collected by searching the chemical composition literature of Ilex asprella root, and these components were imported into SwissADME for ADME screening, with GI absorption as high and Druglikeness greater than or equal to two as Yes. The drug-likeness was greater than or equal to two as Yes as the screening criteria, and the target of the action of Ilex asprella root was predicted in the SwissTargetPrediction database, and the target with a Probability value greater than 0.1 was selected as the predicted target, and the target of the action of Ilex asprella root (GMG) was obtained.

### Collection of influenza-related targets and acquisition of potential therapeutic targets

The keyword "influenza" was used to search the Genecards database (https://www.genecards.org/), Online Mendelian Inheritance in Man (OMIM, https://www.omim.org/^31^, the DisFeNET database (https://www.disgenet.org/), Therapeutic Target Database (TTD, http://db.idrblab.net/) and DrugBank database (https://go.drugbank.com/) to search and screen for disease targets of influenza. relevant targets for influenza.

### Acquisition of *GHQWG* targets in flu

To clarify the mechanism of action of drug targets and disease targets at the protein level, drug genes, and disease genes were submitted to the Venn diagram tool (http://bioinformatics.psb.ugent.be/webtools/Venn/^32^, it was possible to further achieve the goal of removing the repeated targets among *GHQWG* and flu. Then, the intersection of *GHQWG*-related targets and flu-related targets was then screened for common targets^33^.

### Analyses of the protein–protein interaction network and hub targets

The protein–protein interaction (PPI) network helps to better understand the biological mechanisms involved in target-related pathogenesis at the protein level. Thus, the STRING 11.0b database (https://string-db.org/) was used to construct the PPI network and receive hub targets. The organism was set to “Homo sapiens” and the minimum required interaction score was 0.4^34^. Subsequently, the PPI network was visualized and analyzed by Cytoscape 3.7.2 software (https://cytoscape.org/). The degree values in the PPI network were calculated by using the NetworkAnalyzer plugin of Cytoscape 3.7.2 software, calculates the degree of each target node of the PPI network through topological analysis, and selects the key target genes in the network according to the degree of the node, and the larger the degree, the greater the role of the gene in the PPI network, and then takes the top 6 proteins as the core targets. The proteins with the top 6 Degree values were selected as the core targets.

### Enrichment analyses for common targets

To study the biological function of potential targets in flu, DAVID (https://david.ncifcrf.gov/) database was used to collect GO analysis and KEGG data^35^. GO analysis is used to screen biological processes (BP), cellular components (CC), and molecular functions (MF)^36^. KEGG enrichment analysis can find important signaling pathways involved in biological processes^37–39^. Subsequently, GO and KEGG data were uploaded to the Bioinformatics (http://www.bioinformatics.com.cn/) platform for visual analysis^40^.

### Molecular docking

Molecular docking can predict the potential therapeutic effects of drug components by analyzing the binding potential between the main components and hub genes^41^. The specific methods were as follows: the 3D structure of the hub genes proteins and the crystal structure of the main components were obtained from the RCSB PDB (http://www.rcsb.org/) and PubChem databases^42^ respectively. PyMOL 1.7.x software was used to remove ligands, dehydrate, and hydrogenate the hub genes proteins^43^. Then, the hub genes proteins and main components were transformed into PDBQT format by AutoDock Tools 1.5.6 software. AutoDock Tools 1.5.6 was used to construct a crystal structure docking grid box for each target. Then the molecules with the lowest binding energy for each active compound in the docking conformation were allowed for semi-flexible docking by comparing with the original ligands and intermolecular interactions. Box center coordinates and size of the box were determined for evaluating the interaction and the “Number of GA Runs” was set to 50^44^. Finally, the docking results were visualized with PyMOL software.

### Molecular dynamics simulation

To analyze the binding affinities of protein targets and active compounds obtained by molecular docking, a 100 ns atomistic molecular dynamics (MD) simulation was performed using the AMBER20 software package^45,46^. The ff14SB force field parameter was used for the proteins, and the general AMBER force field (gaff) was used for the active ingredients. The electrostatic potentials were calculated at the B3LYP/6-31G level using Gaussian09 and the atomic partial charges were assigned using the Antechamber package in AMBER20. After loading the protein, active ingredients were uploaded to the leap module, and hydrogen atoms and antagonist ions were automatically added to neutralize the charge. The TIP3P explicit water model was selected, and periodic boundary conditions were set^47^. The molecular dynamics simulation workflow included four steps: energy minimization, heating, equilibrium, and production dynamics simulation. First, the heavy atoms of proteins (and small molecules) were confined, and 5000 steps (including the 2500-step steepest descent method and the 2500-step conjugate gradient method) were performed on the water molecules to minimize the energy; then, the system was slowly heated from 0 to 300 K within 60 ps. After heating, the system at 60 ps under the NPT ensemble was balanced. Finally, the system was subjected to a molecular dynamics simulation of 100 ns (a total of 50,000 steps) under the NPT ensemble. The Particle Mesh Ewald (PME) methods were used to deal with long-range electrostatic interactions and the SHAKE algorithm was used to restrict the bond to the hydrogen atom. The time-step was set at 2 fs, and the trajectory data were saved every 1 ps. The CPPTRAJ module was used for analyses^48^.

## Results

### Active compounds and potential targets of *GHQWG*

**Figure 2:**
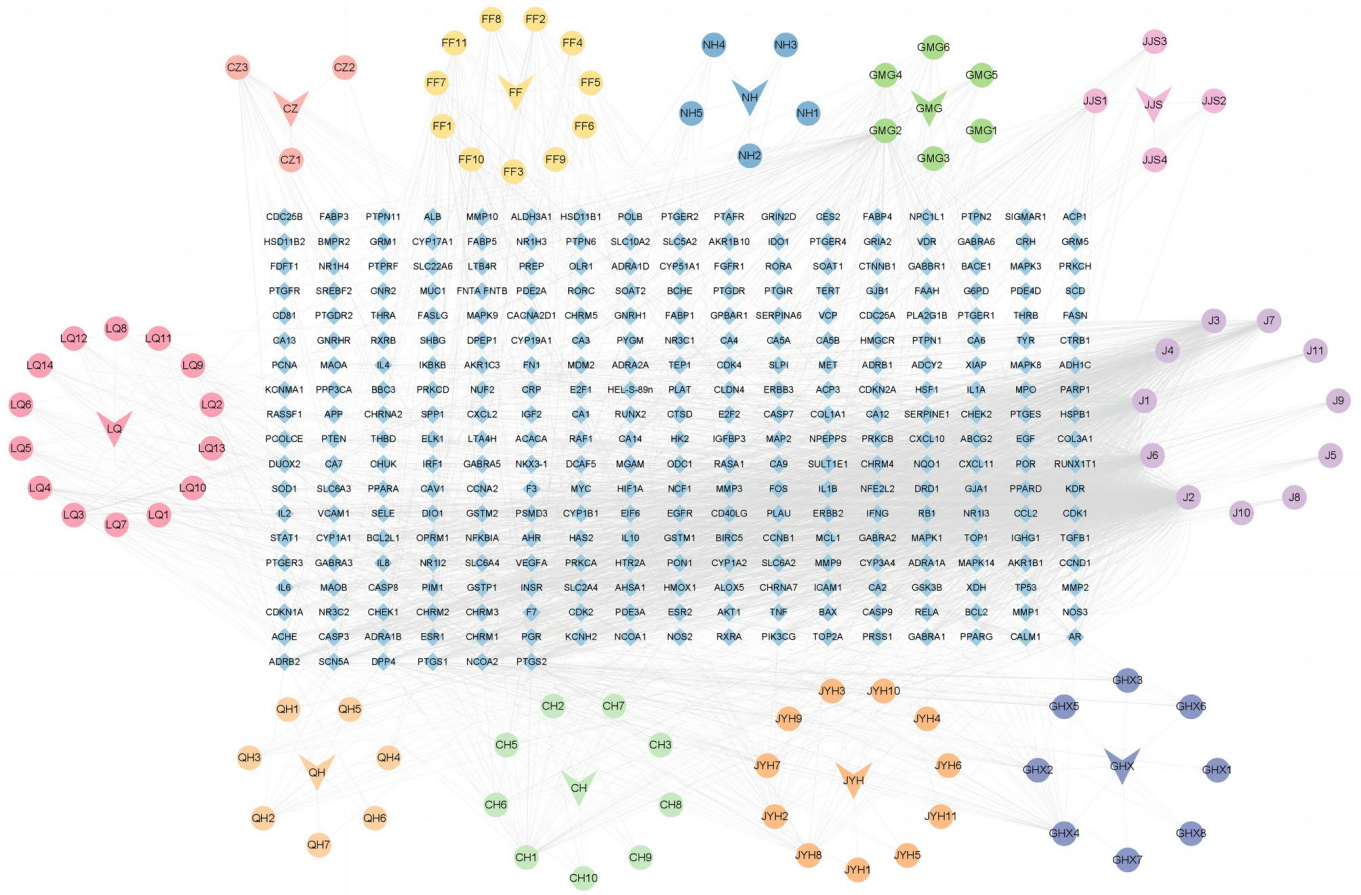
Herb–compound–target network. Blue arrows represent the herbs in *GHQWG*, yellow circles node are QH compounds, green circles node are CH compounds, orange circles node are JYH compounds, deep purple circles node are GHX compounds, light purple circles node are intersection components, pinkish purple circles node are JJS compounds, dark green circles node are GMG compounds, navy blue circles node are NH compounds, bright yellow circles node are FF compounds, red–orange circles node are CZ compounds, powdered circles node are LQ compounds. The edges represent the interaction between compounds and targets, and the node size is proportional to the degree of interaction.

In total, 135 chemical compounds from ten herbs in *GHQWG* were obtained from TCMSP databases and the literature, including 6, 11, 23, 9, 17, 18, 8, 23, 5 and 15 compounds from GMG, GHX, JYH, CZ, CH, FF, JJS, LQ, NH and QH, respectively. After removing redundancy, 90 chemical compounds were identified (Supplementary File Table 1). 312 probable targets of *GHQWG* active chemicals and their related gene symbols were identified by scanning the TCMSP target module (Supplementary File Table 2). The active compounds and common targets were loaded into Cytoscape 3.7.1 to create a herb-compound-target network with 411 nodes and 2134 edges (Fig. 2). The network sheds light on the multiple complicated impacts of GHQWG active chemicals on flu. We determine the key components based on the degree ranking of the active ingredients, and identify the top eight active ingredients with degree ranking as key components. A network analysis showed that quercetin (degree: 585), Kaempferol (degree: 175), Luteolin (degree: 163), β-Sitosterol (degree: 137), wogonin (degree: 127), Stigmasterol (degree: 91), Caffeic Acid (degree: 82), and Isorhamnetin (degree: 33) were key nodes.

### Prediction of drug and disease related targets

**Figure 3:**
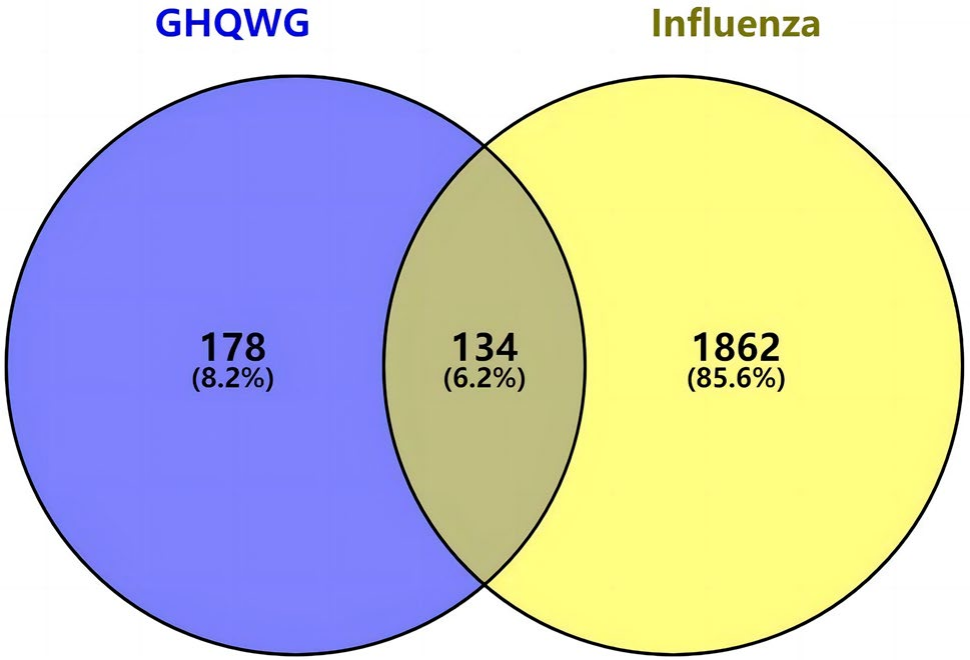
Disease–drug target Venn diagram.

We collect target genes related to flu from five open-source databases, namely, GeneCards (1656), OMIM (5), DisGeNET (314), TTD (17), and DrugBank (232). After checking duplications, 1996 target genes were obtained (Supplementary File Table 3). Using the Venny online mapping tool, 1996 influenza-related targets and 331 *GHQWG* projected targets were loaded. Following mapping, 134 intersection targets of *GHQWG* and influenza were obtained (Fig. 3).

### Construction and analysis of *GHQWG*-flu-related PPI network

**Figure 4:**
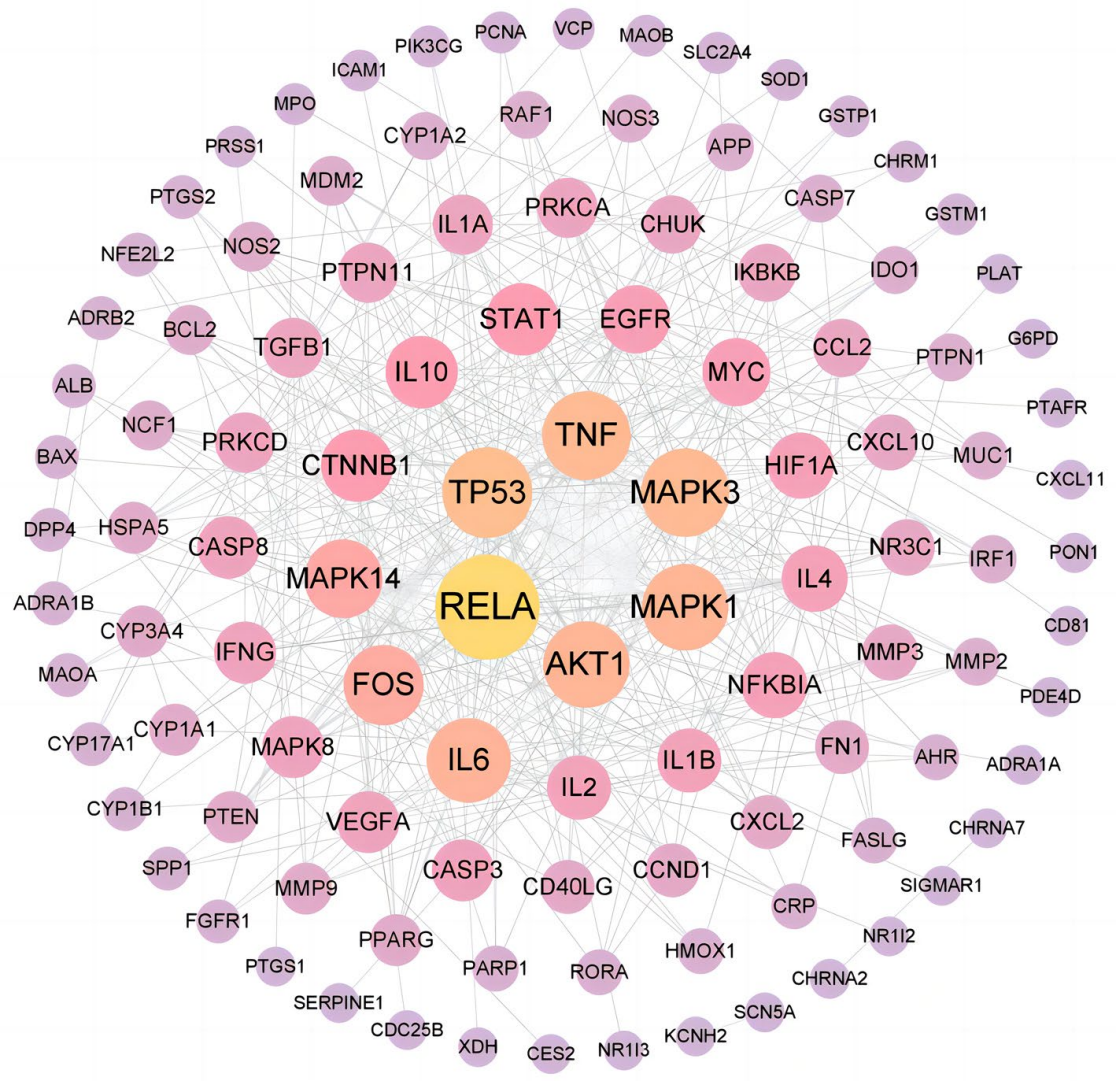
Core target PPI network. As shown in the figure, the darker color of the circle is proportional to its importance in this network.

134 intersection targets were then imported into the STRING platform to build a PPI network, yielding 109 nodes and 397 edges. The size and color of the node denoted the size of the value. The intersection targets were screened using the double median of "Degree," that is, "Degree 24." As a result, the PPI network data from the STRING11.5 database was imported into the Cytoscape 3.9.1 software for visualization (Fig. 4). The PPI analysis revealed that the therapeutic targets of *GHQWG* had many networks and synergistic interactions. Topological analysis showed RELA proto-oncogene, NF-kBr (RELA), tumor protein p53 (TP53), mitogen-activated protein kinase 3 (MAPK3), tumor necrosis factor (TNF), AKT serine/threonine kinase 1 (AKT1), and mitogen-activated protein kinase 1 (MAPK1) occupy the core position in the PPI network and are considered hub genes of *GHQWG* against flu (Supplementary File Table 5).

### Gene Ontology and Kyoto Encyclopedia of Genes and Genomes pathway analysis

**Figure 5:**
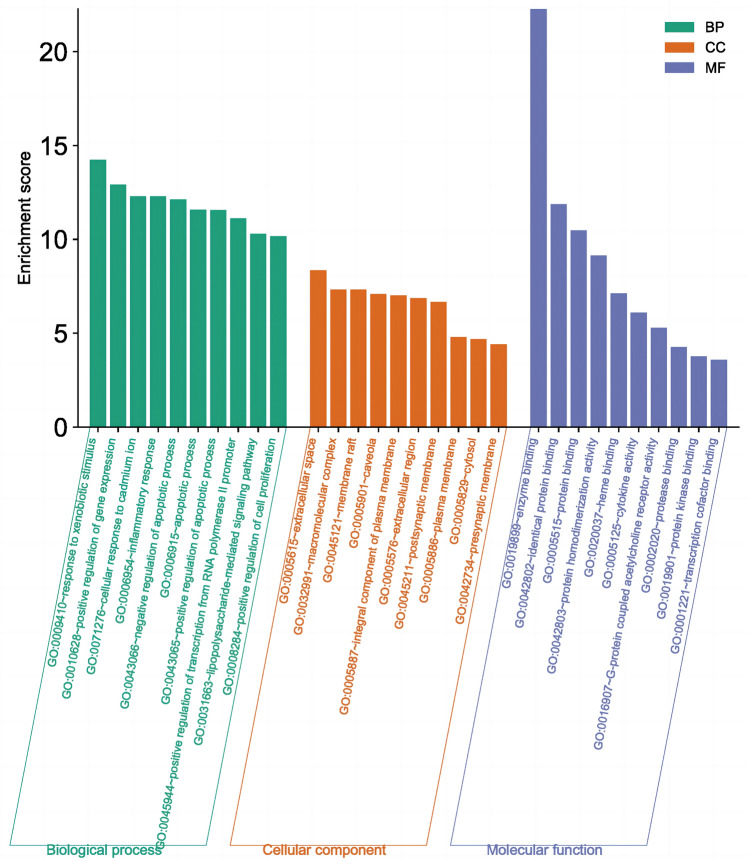
The GO function analyzes the histogram. BP is marked in teal, CC in sienna, and MF in steel blue. The bar graph is obtained by the Bioinformatics Platform.

**Figure 6:**
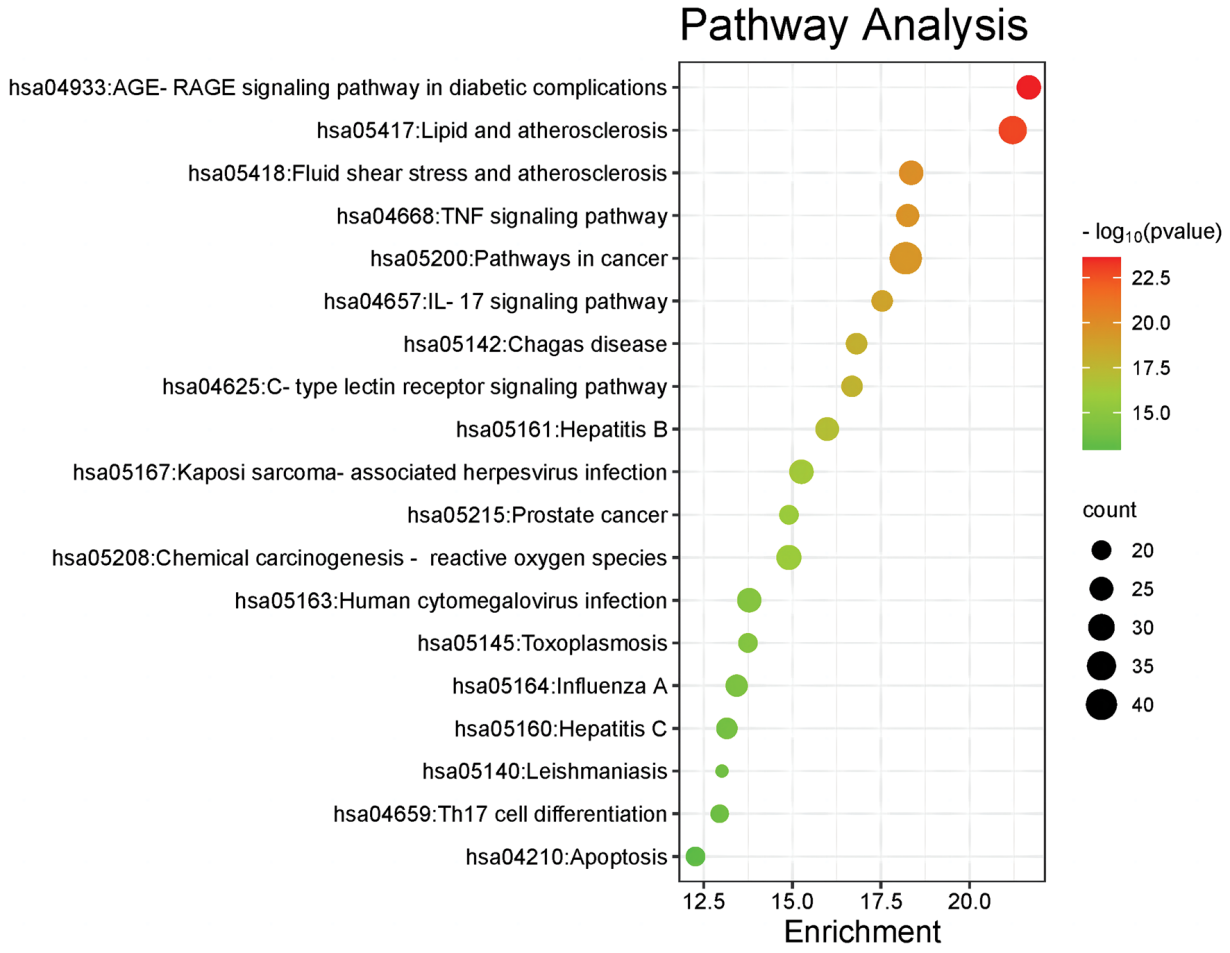
KEGG enrichment bubble diagram of the treatment of flu by GHQWG.

To study the biological activities and pathways of *GHQWG* against flu, GO and KEGG enrichment analysis on common targets were done. On the Metascape platform, the GO function enrichment study of the 134 core targets yielded 888 GO items, comprising 674 "Biological Processes (BP)", 80 "Cellular Components (CC)", and 134 "Molecular Functions (MF)"(Supplementary File Table 6). Based on the P value for visual analysis, the first 15 "Biological Processes" items, 10 "Cellular Components" items, and 10 "Molecular Functions" items were chosen (Fig. 5). Representative BP terms included the response to xenobiotic stimulus, positive regulation of gene expression, cellular response to cadmium ion, inflammatory response, positive regulation of the apoptotic process, positive regulation of transcription from RNA polymerase II promoter, lipopolysaccharide-mediated signaling pathway, positive regulation of cell proliferation, etc. Representative CC terms included extracellular space, macromolecular complex, membrane raft, caveola, an integral component of the plasma membrane, extracellular region, postsynaptic membrane, plasma membrane, cytosol, presynaptic membrane, etc. Representative MF terms included enzyme binding, identical protein binding, protein binding, protein homodimerization activity, heme binding, cytokine activity, G-protein coupled acetylcholine receptor activity, protein kinase binding, transcription cofactor binding, etc. In addition, 174 signal pathways were enriched (Supplementary File Table 7) by KEGG pathway analysis of the core targets using the DAVID platform (Fig. 6) representative pathways included the AGE-RAGE signaling pathway in diabetic complications, Lipid and atherosclerosis, Fluid shear stress and atherosclerosis, TNF signaling pathway, Pathways in cancer, IL- 17 signaling pathway, Chagas disease, C- type lectin receptor signaling pathway, Hepatitis B, Kaposi sarcoma-associated herpesvirus infection, Prostate cancer, Chemical carcinogenesis—reactive oxygen species, Human cytomegalovirus infection, etc. In conclusion, Go and KEGG analysis highlighted that *GHQWG*’s anti-inflammatory, antiviral, and anticancer properties are important targets/pathways in flu treatment.

### Molecular docking

**Figure 7:**
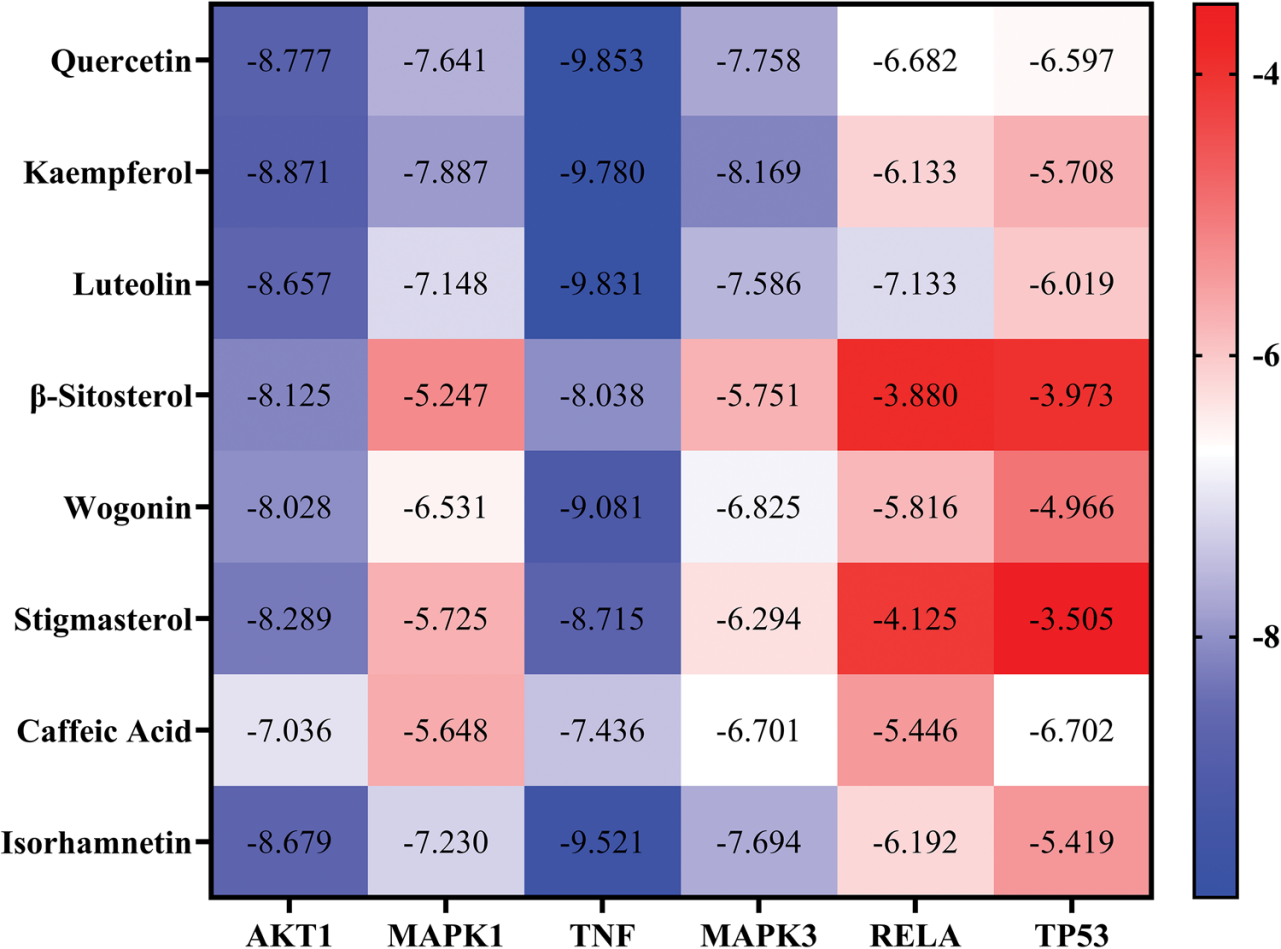
Heat map of binding energies.

In general, it is thought that a docking score of 0 kcal/mol or less suggests that the component and the target can interact spontaneously, a value of − 4.0 kcal/mol or less shows good docking affinity, a value of − 7.0 kcal/mol or less shows strong docking affinity, and the smaller the value, the higher the binding activity^49–51^. The docking targets with the 8 active components with the highest degree of Quercetin, Kaempferol, Luteolin, β-Sitosterol, Wogonin, Stigmasterol, Caffeic Acid, Isorhamnetin, and the top 6 targets of degree are RELA, TP53, MAPK3, TNF, AKT1, and MAPK1 were used for molecular docking analysis. The docking results have shown the main components and hub genes have good binding activity (affinity < − 6.00 kcal/mol) (Fig. 7) and the binding energy of quercetin and hub genes was the lowest, indicating that quercetin had the strongest binding ability. Detailed information on molecular docking is shown in Table 1.

**Table 1:** The molecular docking results of the hub genes with the main components of GHQWG.

Core target	Binding energy (kcal/mol)					
	Quercetin	Kaempferol	Luteolin	β-Sitosterol	Wogonin	Stigmasterol
AKT1	− 8.777	− 8.871	− 8.657	− 8.125	− 8.028	− 8.289
MAPK1	− 7.641	− 7.887	− 7.148	− 5.247	− 6.531	− 5.725
TNF	− 9.853	− 9.780	− 9.831	− 8.038	− 9.081	− 8.715
MAPK3	− 7.758	− 8.169	− 7.586	− 5.751	6.825	− 6.294
RELA	− 6.682	− 6.133	− 7.133	− 3.880	− 5.816	− 4.125
TP53	− 6.597	− 5.708	− 6.019	− 3.973	− 4.966	− 3.505

**Figure 8:**
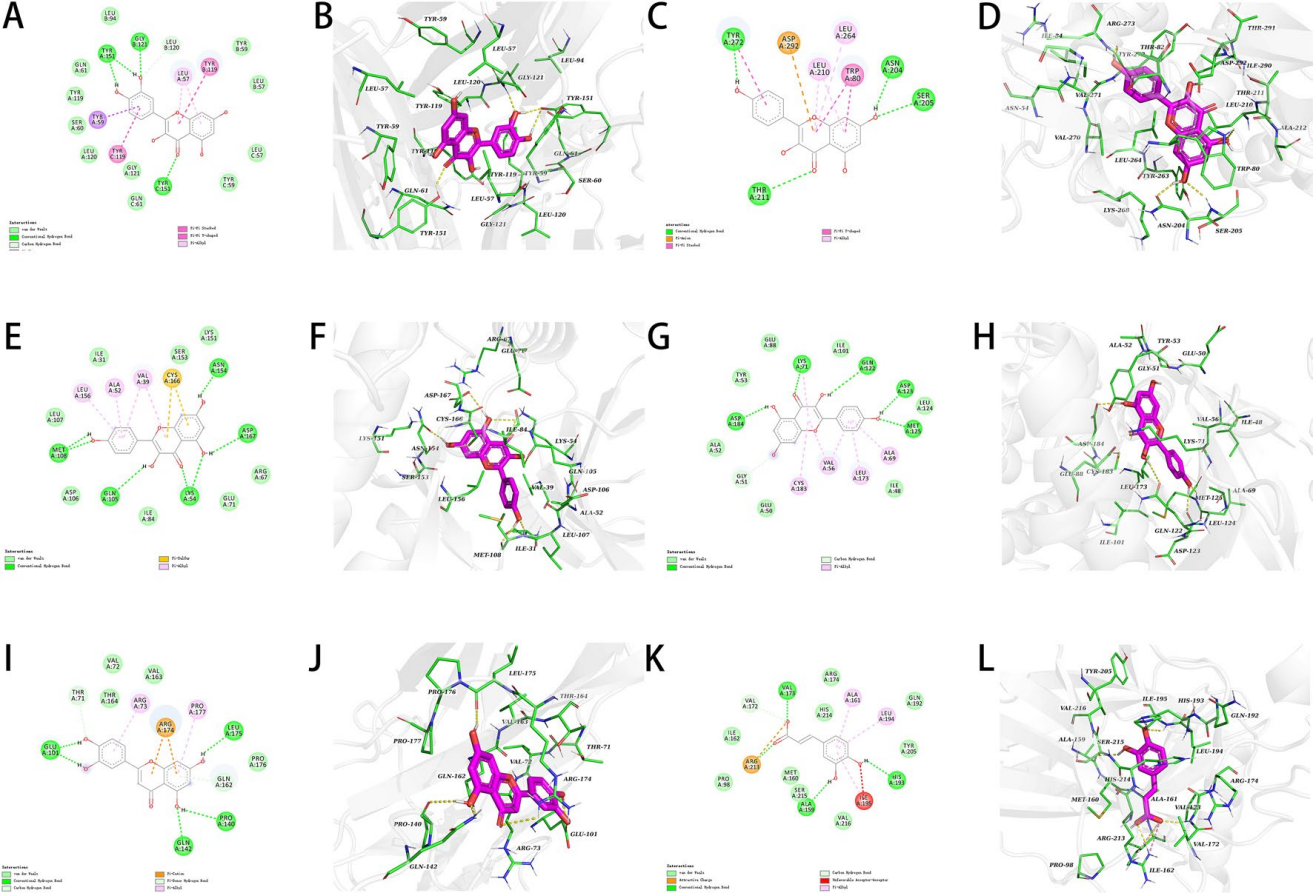
Molecular docking diagram of chemical composition to target: TNF to Quercetin (2D and 3D) (**A**,**B**); AKT1 to Kaempferol (2D and 3D) (**C**,**D**); MAPK1 to Kaempferol (2D and 3D) (**E**,**F**); MAPK3 to Kaempferol (2D and 3D) (**G**,**H**); RELA to Luteolin (2D and 3D) (**I**,**J**); TP53 to Caffeic-Acid (2D and 3D) (**K**,**L**).

PyMoL-1.7.2.1 and Discovery Studio 2020 were used to depict the compound-target interactions with the greatest free binding energy scores, as well as their mechanisms of binding (Fig. 8A–L). Docking results had free binding energies ranging from − 3.505 to − 9.853 kcal/mol, indicating stable binding. The free binding energy of TNF with quercetin was − 9.853 kcal/mol. Binding affinities were attributed to hydrogen bonding with TYR-199 residues van der Waals forces with LEU-94, LEU-120, TYR-59, LEU-57, GLN-61, GLY-121, LEU-120, SER-60, TYR-119 and GLR-61 residues, as well as hydrophobic interactions with LEU-57 residues of TNF (Fig. 8I,J).

### Molecular dynamics simulation

Molecular dynamics simulations can be used to understand the stability of protein–ligand complexes. Based on the results of molecular docking, we selected the six compounds with the highest free binding energy scores as the targets of molecular dynamics simulations, namely AKT1-Kaempferol, MAPK1-Kaempferol, MAPK3-Kaempferol, RELA-Luteolin, TNF-Quercetin, and TP53-Caffeic Acid were subjected to simulation analyses of molecular dynamics for 100 ns to assess their motion, trajectory, structural features, binding potential, and conformational changes.

**Figure 9:**
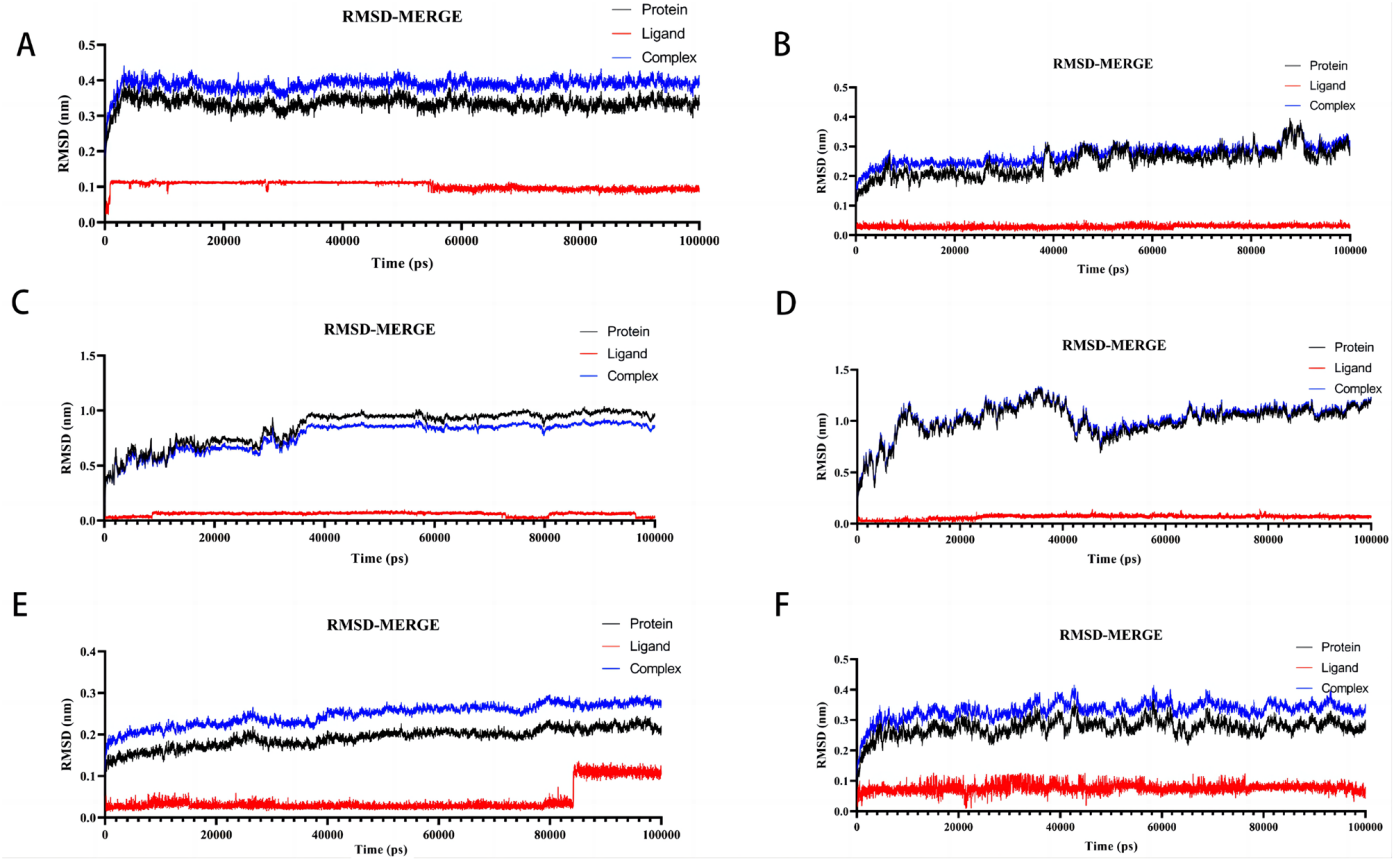
Root-mean-square deviation (RMSD) plots during of molecular dynamics simulation. (**A**) The RMSD of AKT1-Kaempferol. (**B**) The RMSD of MAPK1-Kaempferol. (**C**) The RMSD of MAPK3-Kaempferol. (**D**) The RMSD of RELA-Luteolin. (**E**) The RMSD of TNF-Quercetin. (**F**) The RMSD of TP53-Caffeic Acid.

The root mean square deviation (RMSD), which measures the degree of atom position departure from the initial position, is a useful indicator of the conformational stability of proteins and ligands. An improved conformational stability is shown by a decreased deviation. The complexes' variations in RMSD values were examined. The RMSD of the AKT1–Kaempferol complex varied early on and stabilized after 0.4 nm, as seen in Fig. 9A. Although the MAPK1–Kaempferol complex's RMSD trajectory varied between 86,000 and 90,000 ps and was largely smooth the remainder of the period (Fig. 9B), it did so. The trajectory became steady for the MAPK3–Kaempferol complex at 1 nm (Fig. 9C). The RELA–Luteolin complex's RMSD fluctuated early on before stabilizing at 48,000 ps (Fig. 9D). RMSD of the TNF-Quercetin combination has been stabilized overall (Fig. 9E). In the beginning, the RMSD of the TP53–Caffeic Acid complex varied until stabilizing at 0.4 nm (Fig. 9F).

The active pockets of small molecules and proteins were found to be in a stable condition based on the RMSD values for the ligand and pocket. This shows that the protein's structure does not change considerably following the interaction with the small molecule ligand, and the combination is reasonably stable.

Together, these findings resoundingly validate the docking results. Temperature and pressure have no discernible impact on the structural conformation.

#### The stability of the target protein at the residue level

**Figure 10:**
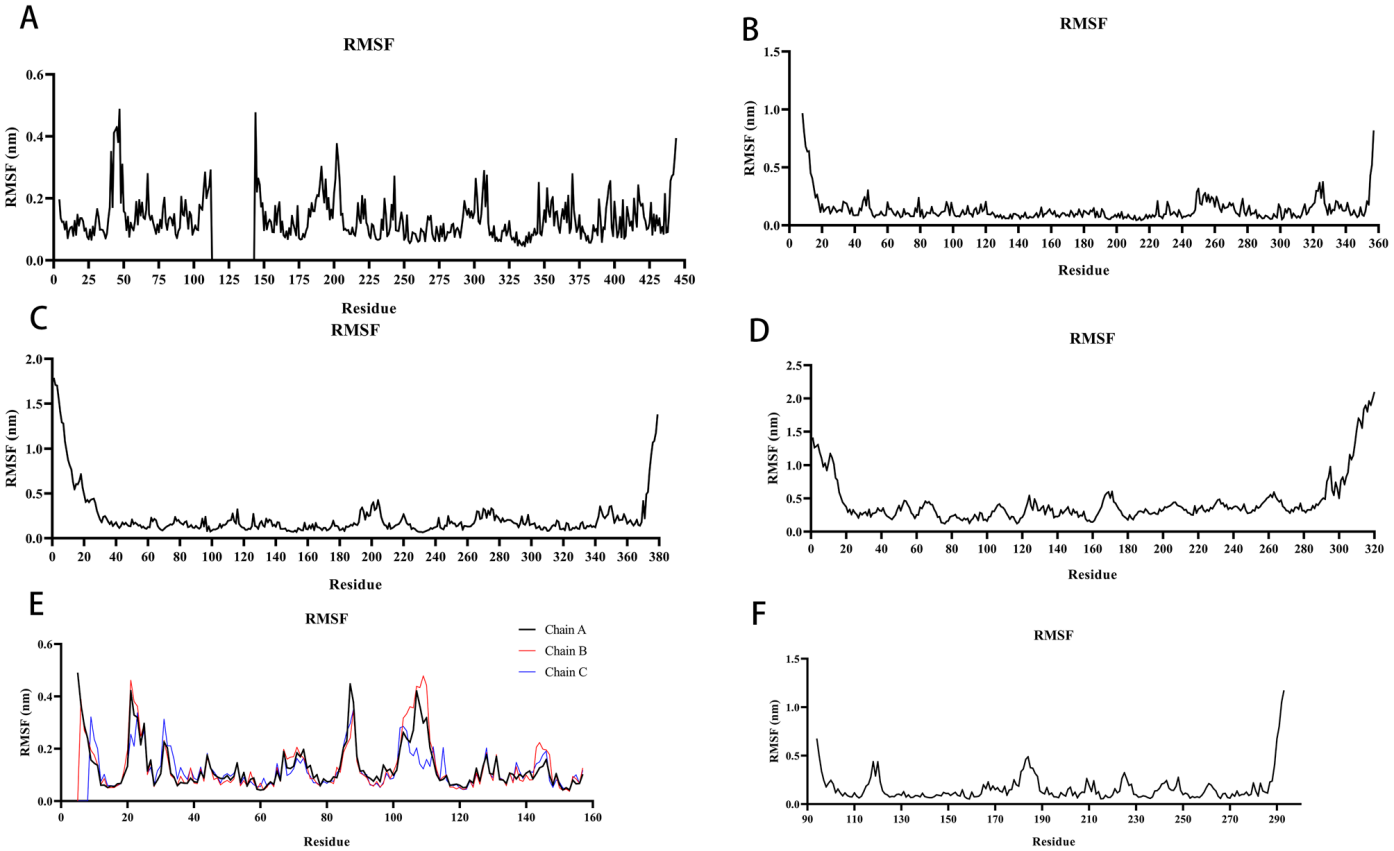
MD simulates RMSF trace values of protein–ligand complexes. (**A**) The RMSF of AKT1-Kaempferol. (**B**) The RMSF of MAPK1-Kaempferol. (**C**) The RMSF of MAPK3-Kaempferol. (**D**) The RMSF of RELA-Luteolin. (**E**) The RMSF of TNF-Quercetin. (**F**) The RMSF of TP53-Caffeic Acid.

The vibrations of each residue following chemical binding were examined as root mean square fluctuations (RMSF) in order to investigate the local fluctuations of macromolecular proteins at the residue level. RMSF can be used in molecular dynamics simulations to depict the flexibility of proteins. The medication typically has the function of stabilizing the protein and exerting the effect of enzymatic activity by binding to the protein and causing a reduction in its flexibility. Protein amino acid flexibility and motion intensity are described by RMSF throughout the simulation. The medication attaches to the protein, stabilizing it and activating enzymes, as seen in the picture, which generally makes the protein less flexible. All compounds show the same tendency when compared to the respective reference ligand, i.e., they cause some fluctuation in the same protein region (Fig. 10A–F). However, all compounds generally have high rigidity and structural stability.

#### Analysis of the radius of gyration

**Figure 11:**
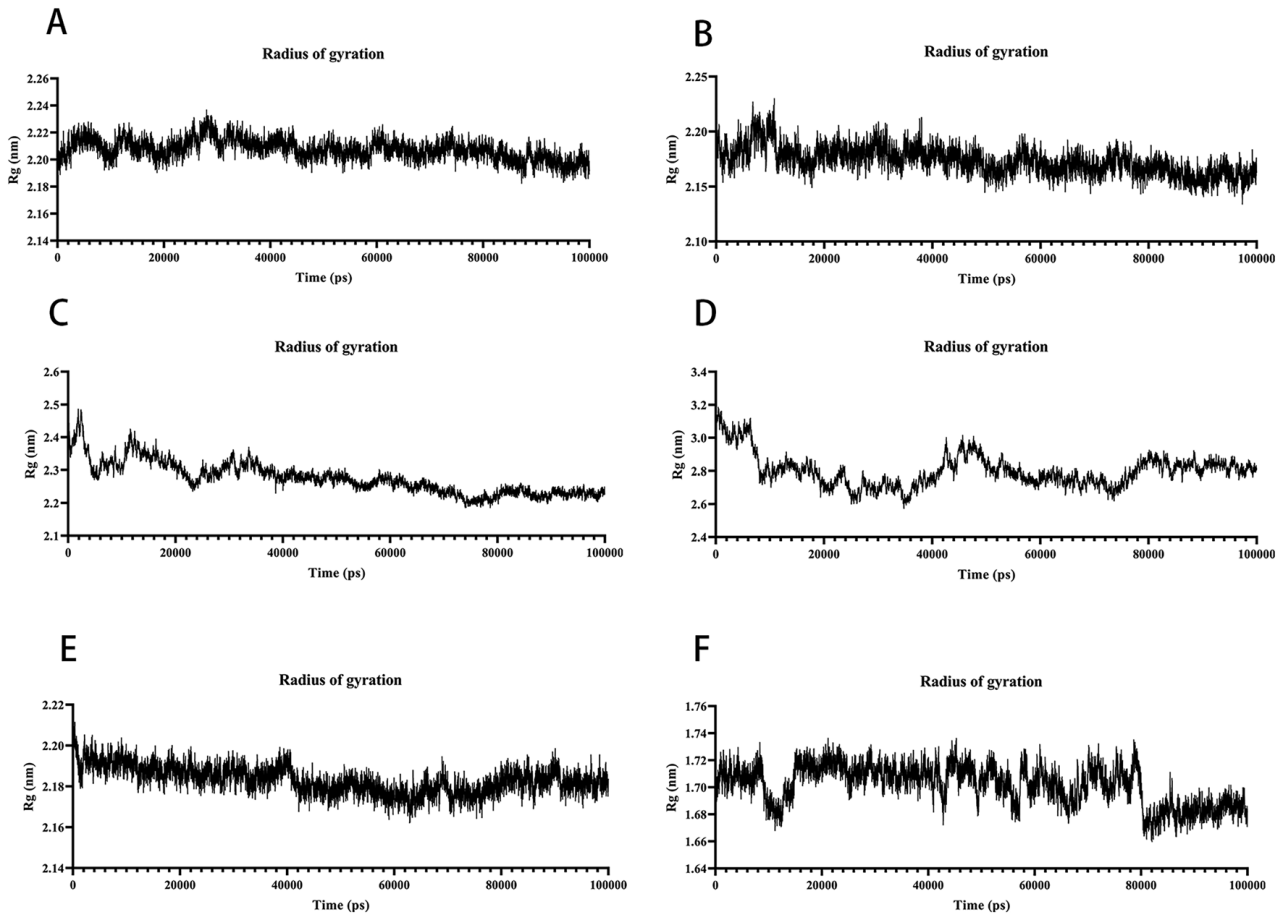
MD simulates Rg trace values of protein–ligand complexes. (**A**) The Rg of AKT1-Kaempferol. (**B**) The Rg of MAPK1-Kaempferol. (**C**) The Rg of MAPK3-Kaempferol. (**D**) The Rg of RELA-Luteolin. (**E**) The Rg of TNF-Quercetin. (**F**) The Rg of TP53-Caffeic Acid.

The radius of gyration (Rg) represents the compactness of the embodiment and can indicate the degree of system binding. Rg, as indicated in the figure, can be used to reflect the protein structure's compactness. Throughout the simulation, the structural dynamics of AKT1–Kaempferol, MAPK1–Kaempferol, and TNF–Quercetin complexes remained relatively stable (Fig. 11A,B,E). MAPK3-Kaempferol, RELA-Luteolin, and TP53-Caffeic Acid fluctuated, and the Rg value was biased (Fig. 11C,D,F), indicating that kaempferol was strongly bound to most proteins. Meanwhile, the Rg of AKT1–Kaempferol complex, MAPK1–Kaempferol complex, MAPK3–Kaempferol complex and TNF–Quercetin complex were stabilised at 2.16–2.25 nm; that of RELA–Luteolin complex was stabilised at 2.80 nm and that of TP53-Caffeic Acid was stabilised at 1.67 nm. The Rg of the RELA–Luteolin complex eventually stabilised at 2.80 nm, and that of the TP53–Caffeic Acid complex eventually stabilised at 1.67 nm.

#### Hydrogen bond analysis

**Figure 12:**
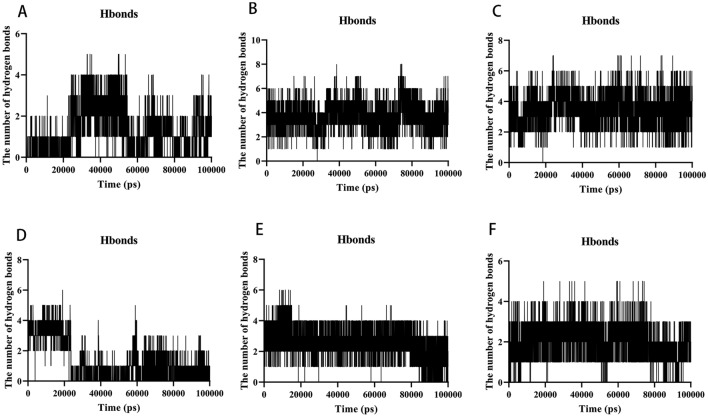
MD simulates the number of hydrogen bonds (HBond) of protein–ligand complexes. (**A**) The HBond of AKT1-Kaempferol. (**B**) The HBond of MAPK1-Kaempferol. (**C**) The HBond of MAPK3-Kaempferol.(D)The HBond of RELA-Luteolin.(E)The HBond of TNF-Quercetin. (**F**) The HBond of TP53-Caffeic Acid.

Because hydrogen bonding is one of the most powerful non-covalent binding interactions, understanding the binding affinity between ligands and proteins is critical. The results indicated that the hydrogen bond numbers for the AKT1–Kaempferol, MAPK1–Kaempferol, MAPK3–Kaempferol, RELA–Luteolin, TNF–Quercetin, and TP53–Caffeic Acid complexes were 0–6, 0–10, 0–8, 0–8, 0–8, and 0–6, respectively (Fig. 12A–F). The number of hydrogen bonds produced by all protein–ligand complexes remains constant throughout the simulation. Furthermore, the presence of persistent amino acid residues at the active site contributes to the complex's overall structural stability.

#### Analysis of solvent accessible surface area

**Figure 13:**
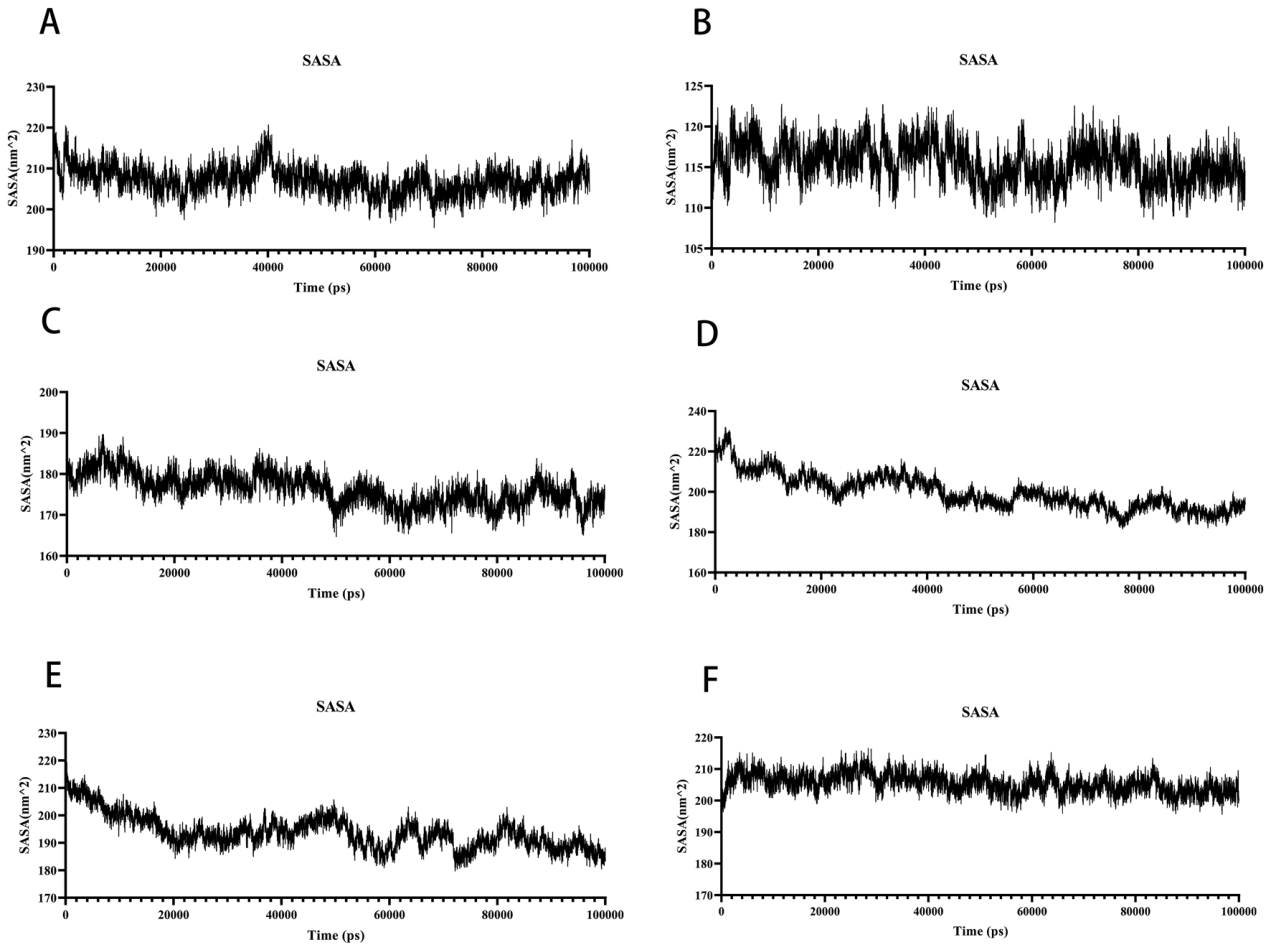
MD simulates SASA of protein–ligand complexes. (**A**) The SASA of AKT1-Kaempferol. (**B**) The SASA of MAPK1-Kaempferol. (**C**) The SASA of MAPK3-Kaempferol. (**D**) The SASA of RELA-Luteolin. (**E**) The SASA of TNF-Quercetin. (**F**) The SASA of TP53-Caffeic Acid.

The interface enclosed by solvent is used to compute the solvent accessible surface area (SASA)^52^. Because this solvent behaves differently under different conditions, it can be used to examine protein conformational dynamics in a solvent context. The contact area between the six complexes and water is comparable, and the small molecule has little effect on the protein and water effect, the results are shown in (Fig. 13A–F). The SASA values of AKT1–Kaempferol complex, TP53–Caffeic Acid complex, MAPK3–Kaempferol complex and AKT1–Kaempferol complex remained generally unchanged during the simulation, RELA–Luteolin complex decreased from the initial 230 nm^2^ to 195 nm^2^ and TNF–Quercetin complex decreased from the initial 215 nm^2^ to 185 nm^2^ during the simulation, indicating that the protein–protein interactions have little effect on the characterisation and stability of the surface of protein molecules.

## Discussion

Seasonal influenza epidemics with varying degrees of intensity present challenges to public health in the early twenty-first century, where influenza remains a significant cause of mortality^5^. Antiviral therapy can be difficult to use because of mutations and the emergence of resistance^53^. According to research, symptoms of influenza virus infection range from mild upper respiratory tract infections with symptoms like fever, sore throat, runny nose, cough, headache, muscle pain, and fatigue to severe and occasionally fatal pneumonia caused by the influenza virus or secondary bacterial infection of the lower respiratory tract^54^. In some instances, infection with the influenza virus can result in a variety of non-respiratory consequences that can affect the heart, central nervous system, and other organ systems^55,56^, Human mortality rates and the severity of infection are frequently correlated with a lack of pre-existing immunity^57^. TCM has been utilized for disease prevention and treatment based on systematic multi-target/multi-component techniques for thousands of years^58^. TCM has long been utilized as a treatment method for illnesses connected to the flu in particular^59^.

Based on clinical and experimental research, *GHQWG* is a possible therapy for influenza. The bioactive chemical components and the underlying mechanism of *GHQWG*'s anti-flu therapeutic actions are still unknown. As a result, we employed molecular docking and a network pharmacology technique to pinpoint possible *GHQWG* targets and modes of action in flu.

Using the ADME criteria, we screened 90 active *GHQWG* molecules from the TCMSP databases. Then, through GeneCards, OMIM, DisGeNET, TTD, and DrugBank, we collected 1996 targets for influenza illness. There were 134 potential *GHQWG*-flu targets found in all. Based on the network pharmacology analysis, the top eight active compounds of *GHQWG* were screened, which were quercetin, kaempferol, luteolin, beta-sitosterol, wogonin, Stigmasterol, Caffeic Acid, and isorhamnetin respectively. Previous studies have proven that these chemicals are effective at treating the flu. The initial stage of influenza virus infection, which includes viral attachment, endocytosis, and viral-cell fusion, was inhibited by quercetin^60^. Kaempferol can prevent the replication and autophagy caused by the influenza A virus^61^. Luteolin reduces influenza virus production. A virus in vitro by preventing the production of the coat protein I complex^62^. Strong In Vitro Antiviral Activity of β-Sitosterol Against Influenza A Viruses^63^. Wogonin has strong anti-influenza properties that are controlled by AMPK activation^64^. Treatment with isorhamnetin can minimize the production of reactive oxygen species (ROS) brought on by viruses, block the acidification of cytoplasmic lysosomes and the lipidation of microtubule-associated protein 1 light chain 3-B (LC3B), and stop influenza viruses from replicating^65^. Studies have shown that Stigmasterol is the major anti-hemagglutinin binding component and inhibits the spread of influenza virus by inhibiting the activity of influenza virus neuraminidase and preventing the release of viral particles from infected cells^66^. Therefore, based on the above findings, we can find that the active ingredients of *GHQWG* have an inhibitory effect on influenza virus.

The results of the GO functional analysis demonstrated that a variety of biological processes, including the response to xenobiotic stimulus, positive regulation of gene expression, cellular response to cadmium ion, inflammatory response, positive regulation of the apoptotic process, positive regulation of transcription from RNA polymerase II promoter, lipopolysaccharide-mediated signaling pathway, positive regulation of cell proliferation, etc., were connected to the effects of *GHQWG* in influenza. Influenza A virus-induced IFN-alpha/beta is vital in the host's antiviral protection by activating the expression of antiviral Mx, PKR, and oligoadenylate synthetase genes. IFN-alpha/beta increases T cell survival as well, upregulates IL-12 and IL-18 receptor gene expression and together with IL-18 stimulates NK and T cell IFN-gamma production and the development of Th1-type immune response^67^. The targets in this study were enriched in immune-related and inflammatory pathways, such as IL-17 signaling pathways, C-type lectin receptor signaling pathways, and TNF signaling pathways. Previous research has shown that inhibiting intraepithelial TNF- signaling prevents CD8 T-cell-mediated lung injury during influenza infection^68^. In a clinical study, it was found that children with influenza A virus pneumonia had higher serum levels of the cytokine interleukin-17 (IL-17)^69^, which is essential for mediating the immune response to extracellular bacteria and fungus in the lung^70^. Results from an in vivo study revealed that compared to infected wild-type controls, H5N1-infected IL-17 knockout (KO) mice lose weight much more quickly, exhibit more pronounced lung immunopathology, and pass away much sooner. Additionally, B cell density in the lung was drastically decreased in IL-17 KO mice following viral infection. In cultured B cells from IL-17 KO mice, chemokine-mediated migration was significantly reduced. These findings demonstrate that IL-17 is crucial for promoting the migration of B cells to the site of mouse lung influenza virus infection^70^. Influenza mouse lung injury is lessened by epithelial TNF signaling pathway modification because it lowers epithelial chemokine expression and lung inflammatory infiltration^71,72^. TNF triggers the expression of inflammatory genes, which directly triggers inflammatory responses, but it also indirectly triggers cell death, inflammatory immune responses, and the development of diseases^73^.

Furthermore, molecular docking was performed to examine six major target proteins (RELA, TP53, MAPK3, TNF, AKT1, and MAPK1) and active chemicals obtained from TCMSP, including quercetin, Kaempferol, Luteolin, β-Sitosterol, wogonin, Stigmasterol, Caffeic Acid, and Isorhamnetin. Binding affinities for docking data ranged from − 3.880 to − 9.853 kcal/mol, indicating that all of the targets may be capable of docking with active molecules. Among the six target proteins, TNF and AKT1 had the lowest binding affinities. quercetin, kaempferol, and luteolin demonstrated good binding activity to the targets, implying that these chemicals may contribute to *GHQWG*'s therapeutic benefits in flu. More experimental research is needed, however, to confirm and examine the hypothesized targets and regulatory processes.

Finally, we selected the six compounds with the highest free binding energy scores as the targets of molecular dynamics simulations, namely AKT1-Kaempferol, MAPK1-Kaempferol, MAPK3-Kaempferol, RELA-Luteolin, TNF-Quercetin, and TP53-Caffeic Acid were subjected to simulation analyses of molecular dynamics to further verify it. The results showed that kaempferol, luteolin, quercetin, and caffeic acid were able to tightly dock to the individual targets and quickly reached steady state.

It should be noted that there were some restrictions on this study. The reliability and accuracy of the predictions were therefore dependent on the quality of the data because both bioactive chemicals and target information were first gathered from the literature and databases. Second, this study employed a data mining methodology, and additional research using clinical trials and animals is required to validate the results.

## Conclusion

For the treatment of the flu, this is the first time that bioinformatics techniques like network pharmacology, molecular docking, and molecular dynamics modeling have been used to systematically study the pharmacological and molecular mechanism of action of *GHQWG*. These bioinformatic and computational investigations showed that the primary constituents of *GHQWG* with therapeutic actions against the flu may include quercetin, kaempferol, kuteolin, β-sitosterol , wogonin, stigmasterol, caffeic Acid, and Isorhamnetin. Additionally, *GHQWG* can treat the flu by lowering pathologic harm, inflammatory reactions, and oxidative stress via several channels, including TNF and IL-17.

Overall, the present study concentrated on the multi-component and multi-pathway nature of *GHQWG* and its mode of action. These results are anticipated to guide the use of *GHQWG* and its continued development for the treatment of flu.

## Strengths and limitations

Notably, our study provided some fresh insights into *GHQWG* in flu therapy and revealed feasible biochemical pathways and potential pharmacological targets of *GHQWG* for the first time. However, a few difficulties with our study's limitations must be addressed. Because the findings of this study were not confirmed in genuine flu patients, future confirmation of these findings will necessitate the recruitment of actual flu patients. Second, additional in vivo and in vitro research are required to confirm the predicted mechanisms and pharmacological targets in order to confirm the potential therapeutic applicability of *GHQWG* for flu.

### Supplementary Information

Supplementary Table S1.

Supplementary Table S2.

Supplementary Table S3.

Supplementary Table S4.

Supplementary Table S5.

Supplementary Table S6.

Supplementary Table S7.

## Data Availability

The datasets of the current study are available in public database from SwissADME database (http://www.swissadme.ch/), TCMSP (https://tcmspw.com/tcmsp.php), PubChem (https://pubchem.ncbi.nlm.nih.gov/), Swiss Target Prediction (http://www.swisstargetprediction.ch/), Genecards database (https://www.genecards.org/), Online Mendelian Inheritance in Man (OMIM, https://www.omim.org/), the DisFeNET database (https://www.disgenet.org/), Therapeutic Target Database (TTD, http://db.idrblab.net/) and DrugBank database (https://go.drugbank.com/) , Venn diagram tool (http://bioinformatics.psb.ugent.be/webtools/Venn/), STRING 11.0b database (https://string-db.org/) Cytoscape 3.7.2 software (https://cytoscape.org/), DAVIDdatabase (https://david.ncifcrf.gov/), Bioinformatics (http://www.bioinformatics.com.cn/), and PDB (http://www.rcsb.org/), Supplementary files are provided for compound targets, disease targets, intersection targets, the putative targets of *GHQWG* against flu, PPI information and, GO information, and KEGG information. The data used to support the findings of this study are available from the corresponding author upon reasonable request.

## Abbreviations

*GHQWG*: *Ganghuo Qingwen Granules (20g/time, 3 times/day)*
Flu: Influenza
TCM: Traditional Chinese medicine
TCMSP: Traditional Chinese Medicine Systems Pharmacology Database
DL: Drug-likeness
OB: Oral bioavailability
PPI: Protein–protein interaction
OMIM: Online Mendelian Inheritance in Man
TTD: Therapeutic Target Database
PPI: Protein–protein interaction
GO: Gene Ontology
KEGG: Kyoto Encyclopedia of Genes and Genomes
BP: Biological processes
CC: Cellular components
MF: Molecular functions
